# Unmet supportive care needs and associated factors among young adult cancer patients in Japan

**DOI:** 10.1186/s12885-020-07721-4

**Published:** 2021-01-05

**Authors:** Masako Okamura, Maiko Fujimori, Ayako Sato, Yosuke Uchitomi

**Affiliations:** 1 Division of Behavioral Science Research, Behavioral Sciences and Survivorship Research Group, Center for Public Health Sciences, National Cancer Center, 5-1-1 Tsukiji, Tokyo, 104-0045 Japan; 2 Innovation Center for Supportive, Palliative and Psychosocial Care, National Cancer Center Hospital, Tokyo, Japan

**Keywords:** Cancer patient, Supportive care, Survivor, Unmet needs, Young adult

## Abstract

**Background:**

Young adult cancer patients often face unique challenges and have potential unmet needs. This study aimed (1) to describe unmet supportive care needs among young adults with cancer in Japan, and (2) to identify its associated factors.

**Methods:**

In a cross-sectional web-based survey, 206 young adults with cancer were assessed for supportive care needs. Multiple regression analysis examined whether demographics, clinical variables and social support were associated with unmet supportive care needs.

**Results:**

A total of 206 patients (180 female) with a mean age of 33.7 years (SD = 4.3, range: 22–39) participated. One hundred and fifty-eight participants (76.7%) reported at least one unmet supportive care needs. The top 20 unmet needs included 9 of the 10 psychological needs, 3 of the 5 physical and daily living needs, 8 of the 11 health system and information needs and 1 of the 5 sexuality needs. Multiple regression analysis revealed that perceived poorer PS, experience of change in work/school after a cancer diagnosis and poor social support were significantly associated with higher supportive care needs. The total score of supportive care needs was significantly associated with both psychological distress and QOL.

**Conclusions:**

More than 70% of young adult cancer patients reported unmet supportive care needs and most of those were psychological needs. The findings suggest potential opportunities for intervention in addressing psychological needs rather than physical and information needs.

## Background

Adolescence and young adults (AYAs) with cancer between 15 and 39 years of age are complex and vulnerable as a result of the intersection of disease and developmental stage [1]. AYAs are in various challenging situations related to physical and cognitive development, identity, body image, autonomy, and employment [2, 3]. Nearing the end of or having completed high school, young people turn their attention to employment, further formal education, and establishing an independent living situation and intimate relationships with peers [4]. Young adults are in a phase of life marked by change and upheaval [5]. In addition to providing high quality treatment, when considering support based on these unique characteristics and developing age-specific supportive care resources, it is important to understand their supportive care needs and associated factors.

Previous studies in the United States and Australia showed that AYAs with cancer have unmet information, service and supportive care needs [4, 6–13]. According to a systematic review conducted by Galán et al. [14], the most common needs for AYA cancer survivors are “individualized information and advice”, “counseling and psychological support” and “social support, and social relationship”. AYA cancer survivors tend to seek social support from their families and friends, not their doctors [14]. These results are different from those reported studies on adults [15]. Hoekstra et al. [15] found that being able to talk about impact of the disease, getting medical help for problems not related to cancer and obtaining information about their illness from their doctors were all key factors for adults. Moreover, support and access to specialists (e.g. psychologists) is highly valued by young people [14] but is not common in older cancer survivors. Unmet information and service needs for AYAs with cancer were associated with sex, age, age of first diagnose, race, performance status (PS), marital status, chemotherapy, depression, anxiety and QOL [4, 7–10, 12, 13]. Though psychological distress, impaired physical functioning, cognitive dysfunction and financial hardship occur during treatment and many years after, supportive care needs could change during the transition process from active treatment to survivorship [16]. It is important to investigate the factors associated with unmet needs in order to consider the support of AYA cancer patients.

AYAs with cancer are 2.5% of all cancer patients in Japan [17]. As it’s relatively low rate compared to the other generations, little is known about their unmet needs and associations between various sociodemographic, medical and psychosocial variables and unmet needs among AYAs with cancer in Japan. Therefore, the current study aimed (1) to describe unmet supportive care needs among AYAs with cancer in Japan, and (2) to identify its associated factors.

## Methods

### Participants and procedure

The survey company (Macromill, Ltd.) recruited potential participants from a pool of 10 million who were registered in a national database and sent questionnaires to them online over 5 days in April 2017. Eligible participants were those with cancer followed in an outpatient clinic and aged 16–39 years old at the time of survey. The 15–39 age range is the standard used by the National Cancer Institute in the United States. According to the Ethical Guidelines for Medical and Health Research Involving Human Subjects formulated by the Ministry of Health, Labor and Welfare of Japan, minors who have graduated from junior high school or are 16 years of age or older can be judged to have sufficient judgment ability regarding the conduct of the study. For these reasons, we set the eligibility age from 16 to 39 years. Potential participants first read introductory statements that summarized the contents of the questionnaire and explained they could withdraw at any time if they wished so. Responses were considered consent to participate. Because the legal adult age is 20 years old in Japan, those who were 16–19 years old were asked to confirm the consent of their parents. Responses to the questionnaire were voluntary, and confidentiality was maintained throughout all investigations and analyses. This study was approved by the Institutional Review Board and Ethics Committee of the National Center for Neurology and Psychiatry, Japan, and was conducted in accordance with the principles laid down in the Helsinki Declaration.

### Measures

#### Supportive care needs: The short-form Supportive Care Needs Survey questionnaire (SCNS-SF34)

The SCNS-SF34 is a 34-item self-report questionnaire. It measures perceived needs of cancer patients in five domains: psychological (10 items), health system and information (11 items), physical and daily living (5 items), patient care and support (5 items) and sexuality (3 items). A 5-point Likert scale is used where 1 corresponds with no need (not applicable), 2 no need (satisfied) and 3, 4 and 5 with low, moderate and high levels of unmet need, respectively. Subscale scores were obtained by summing the individual items. In addition, the total score was obtained by summing all the subscales (range = 34–170). A higher score indicated a higher perceived need. As an alternative use, the scale can be used to obtain information on the presence/absence and number of perceived unmet needs (a rating of 3 or higher was regarded as an unmet need), depending on the researcher’s clinical question. Participants were instructed to circle the number that best describes whether they have needed help with each of the statements in the past month. The validity and reliability of the Japanese version of the SCNS-SF34 have been established [18].

#### Perceived social support: The short-version Multidimensional Scale of Perceived Social Support

It measures social support from family, friends and significant other with seven items. A 7-point Likert scale is used, and a low score for a scale represents poor social support. The validity and reliability of the Japanese version of the short-version Multidimensional Scale of Perceived Social Support have been confirmed [19].

#### Psychological distress: The Kessler Psychological Distress Scale (K6)

The K6 scale is a self-rated six-item questionnaire exploring the frequency of psychological distress during past 30 days. Responses are rated on a five-point scale ranging from “none of the time” (0) to “All the time” (4); scores range from 0 to 24. High scores indicate more severe mental disorders. The validity and reliability of the Japanese version of the K6 have been established [20].

#### Quality of life (QOL): The EuroQol 5 Dimensions 5 Levels (EQ-5D-5L)

The EQ-5D-5L measures five dimensions; mobility, self-care, usual activities, pain/discomfort, and anxiety/depression. A 5-point Likert scale is used and a high score for a scale represents a high level of symptomatology and problems. The validity and reliability of the Japanese version of the EQ-5D-5L have been confirmed [21].

#### Demographic and medical characteristics

The participants were asked their demographic and clinical information, as listed in Table 1. Age, sex and occupational status were obtained from the basic information of the monitoring system.

**Table 1:** Characteristics of the study participants (*N* = 206)

Characteristic		N	%
Age	mean: 33.7 (SD = 4.3)	median: 34.5 (range, 22–39)	
Gender	Female	180	87.4
	Male	26	12.6
Education	< 12 y	86	41.7
	> 12 y	120	58.3
Marital status	Married	102	49.5
	Single or divorced	104	50.5
Household size	Living alone	45	21.8
	Two or more	161	78.2
Having children	None	128	62.1
	Age < 20	76	36.9
	Age > 20	3	1.5
Occupational status	Full-time	65	31.6
	Part-time	51	24.8
	Absence from work	16	7.8
	Housemaker	46	22.3
	Unemployed	23	11.2
	Student	1	0.5
	Others	4	1.9
Change in income status after cancer diagnosis			
	No change	103	50.0
	Decreased	86	41.7
	Increased	17	8.3
Change in work and school life after cancer diagnosis			
	No change	91	44.2
	Work/study hour decreased	20	9.7
	Absence from work/school	50	24.3
	Left work/school	58	28.1
	Changed jobs/schools	22	10.7
	Firing	2	1.0
	Other	5	2.4
Caring for parents	No	192	93.2
	Yes	14	6.8
Performance status^a^	0	147	71.4
	1	52	25.2
	2	4	1.9
	3	2	1.0
	4	1	0.5
Cancer site	Uterus	84	40.8
	Breast	25	12.1
	Thyroid	23	11.2
	Ovary	17	8.3
	Lymphoma	15	7.3
	Leukemia	13	6.3
	Intestine/rectum	13	6.3
	Other	16	7.8
Age of the first diagnosis	< 30	111	53.9
	> 30	95	46.1
Years since diagnosis	< 1 year	33	16.0
	1–5 years	105	51.0
	5–10 years	47	22.8
	> 10 years	21	10.2
Recurrence/metastasis	No	164	79.6
	Yes	15	7.3
	Unknown	27	13.1
Chemotherapy	Never	116	56.3
	Yes (undergoing)	16	7.8
	Yes (completed)	74	35.9

^a^Eastern Cooperative Oncology Group criteria

#### Sample size calculations

We planned to perform a multiple regression analysis to examine the factors related to the supportive care needs, and we calculated that 10 times as many subjects as the number of independent variables would be required. Considering the missing data, the planned number of subjects was 150.

### Statistical analysis

First, we described the prevalence of unmet needs. Second, to identify potential demographic, biomedical, and psychosocial factors associated with a high degree of unmet needs, we conducted a preliminary univariate analysis. In this preliminary analysis, the presence or absence of unmet needs was entered as dependent variables. The presence of unmet needs means that there is more than one item rating of 3 or higher in the SCNS-SF34. Demographic and clinical variables and social support were entered as independent variables. For the univariate analyses, an unpaired *t*-test, Mann-Whitney test and chi-square test were conducted, as appropriate. After the univariate analyses, we used a multiple regression analysis to examine the final factors associated with patients’ unmet needs. The dependent variables were total score (total needs) and the score of each domain. Independent variables with a *p*-value of less than 0.05 in the preliminary univariate analyses were entered into the multiple regression analysis. We entered associated factors (sex, age, age of first diagnose, PS, marital status and chemotherapy) which were reported in previous research [4, 7–9, 11, 12] as independent variables as well. A backward stepwise selection method was used to reduce non-significant variables from the models. To investigate the association between the patients’ supportive care needs and psychological distress and QOL, Pearson’s correlation analysis was conducted. Data were analyzed with the SPSS version 25.0 (IBM). All of the tests were two-tailed, with a *p*-value of < 0.05.

## Results

### Patient characteristics

One thousand and twenty-six cancer patients aged between 16 and 39 were registered in the database in April 2017. Seventy-six percent of the patients were female (776 of 1026). Two hundred and six patients responded and completed the survey (87.4%; 180 female). The participants’ mean age was 33.7 years (SD = 4.3, range: 22–39). The most common type of cancer was uterus (40.8%). Sixteen participants (7.8%) were undergoing chemotherapy. The sociodemographic and clinical characteristics are shown in Table 1.

### Prevalence of unmet supportive care needs

One hundred fifty-eight participants (76.7%) reported at least one unmet supportive care need. The top 20 unmet needs were shown in Table 2. ‘Fears of spreading cancer’ was the commonest, followed by ‘Feeling down and depressed’, ‘Anxiety’, and ‘Concerns about the worries of those close to you’. All of those items were in the psychological domain. The top 20 unmet needs included 9 of the 10 psychological needs, 3 of the 5 physical and daily living needs, 8 of the 11 health system and information needs and 1 of the 5 sexuality needs. The prevalence of the ten most frequent unmet needs was over 40%.

**Table 2:** Top 20 unmet needs among young adult patients (*N* = 206)

Unmet needs	total (*N* = 206)	Gender			Marital status			Children			Chemotherapy			
		Male (*N* = 26)	Female (*N* = 180)	*p*	Married (*N* = 102)	Single (*N* = 104)	*p*	Have (*N* = 76)	None (*N* = 128)	*p*	No (*N* = 116)	Under (*N* = 16)	Done (*N* = 74)	*p*
	(%)	(%)	(%)		(%)	(%)		(%)	(%)		(%)	(%)	(%)	
***Psychological***														
Fears of spreading of cancer	**54.9**	**57.6**	**54.4**	*0.756*	**54.9**	**54.8**	*0.989*	**51.3**	**57.0**	*0.421*	**48.3**	**75.0**	**60.0**	*0.058*
Feeling down or depressed	**51.5**	**61.5**	**50.0**	*0.271*	**44.1**	**58.7**	*0.037* *	**42.3**	**57.0**	0.040*	**48.3**	**75.0**	**51.4**	*0.134*
Anxiety	**48.1**	**53.8**	**47.2**	*0.527*	**42.2**	**53.8**	*0.093*	**39.7**	**53.1**	*0.062*	**40.5**	**75.0**	**54.1**	*0.015* *
Concerns about the worries of those close to you	**46.6**	**53.4**	**45.6**	*0.428*	**41.1**	**51.9**	*0.122*	**42.3**	**49.2**	*0.335*	**39.7**	**68.8**	**52.7**	*0.039* *
Feelings of sadness	**45.1**	**50.0**	**44.4**	*0.595*	**38.2**	**51.9**	*0.048* *	**37.2**	**50.0**	*0.073*	**39.7**	**75.0**	**47.3**	*0.026* *
Uncertainty about future	**44.2**	**53.8**	**42.8**	*0.288*	**36.3**	**51.9**	*0.024* *	**33.3**	**50.8**	*0.014* *	34.5	**68.8**	**54.1**	0.004**
Worry that results of treatment are beyond your control	**41.7**	23.1	**44.4**	*0.039* *	**42.2**	41.3	*0.906*	**41.0**	42.3	*0.870*	**35.3**	**81.3**	40.5	0.002**
Keeping a positive outlook	39.8	**53.8**	37.8	*0.118*	32.6	**47.1**	*0.030* *	28.2	**46.9**	*0.008* **	33.6	**68.8**	**54.1**	*0.020* *
Feelings about death and dying	35.9	38.5	35.6	*0.773*	31.4	40.4	*0.178*	**33.3**	37.5	*0.545*	28.4	50.0	**44.6**	*0.037* *
***Physical and daily living***														
Lack of energy/tiredness	**46.1**	42.3	**46.7**	*0.677*	**40.1**	**51.9**	*0.091*	**37.2**	**51.6**	*0.045* *	**45.7**	**75.0**	40.5	*0.043* *
Work around the home	**42.2**	**50.0**	**41.1**	*0.391*	31.4	**52.9**	*0.002*	30.8	**49.2**	*0.009* **	34.5	**75.0**	**47.3**	*0.005* **
Feeling unwell a lot of the time	36.9	**46.2**	35.6	*0.295*	30.4	**43.3**	*0.055*	23.1	**45.3**	*0.001* **	32.8	56.3	39.2	*0.166*
***Health system and information***														
Being informed about things you can do to help yourself to get well	**40.3**	42.3	**40.0**	*0.823*	**37.3**	**43.3**	*0.379*	**38.5**	41.4	*0.676*	**35.3**	**68.8**	41.9	*0.036* *
Being informed about your test results as soon as feasible	38.4	**46.2**	37.2	*0.381*	**35.3**	41.3	*0.372*	**37.2**	39.1	*0.787*	**35.3**	56.3	39.2	*0.268*
Being given explanation of those tests for which you would like explanations	37.8	**50.0**	36.1	*0.172*	32.4	**43.3**	*0.106*	29.5	43.0	*0.053*	37.9	56.2	33.8	*0.244*
Being informed about cancer which is under control or diminishing	37.3	**46.2**	36.1	*0.322*	31.4	**43.3**	*0.078*	32.1	40.6	*0.217*	33.6	**68.8**	36.5	*0.024* *
Being adequately informed about the benefits and side-effects of treatments before you choose to have them	37.0	**46.2**	35.6	*0.295*	33.3	40.4	*0.294*	**33.3**	39.1	*0.408*	**35.3**	56.3	35.1	*0.248*
Having one member of hospital with whom you can talk to about all aspects of your condition, treatment and followup	36.8	38.5	36.7	*0.859*	31.4	42.3	*0.104*	32.1	39.8	*0.261*	34.5	50.0	37.8	*0.473*
Having access to professional counseling if you, family or friends need it	36.4	42.3	35.6	*0.504*	32.4	40.4	*0.231*	**38.5**	40.6	*0.107*	26.7	62.5	**45.9**	0.002**
Being treated like a person not just another case	35.4	42.3	34.4	*0.433*	28.4	42.3	*0.037* *	29.5	39.1	*0.163*	28.4	62.5	40.5	*0.015* *
***Sexuality***														
Changing in sexual feelings	35.4	26.9	33.9	*0.332*	31.4	34.6	*0.359*	28.2	35.9	*0.428*	**35.3**	25.0	37.8	*0.622*

*p < 0.05, **p < 0.01; chi-squared test

Top 10 unmet needs were showed by bolded numbers for each category

We also showed the top 20 unmet needs in each gender (male/female), marital status (married/single), children (have/none) and chemotherapy (no/under/completed), and the results of the chi-square test (Table 2). Single patients and patients who don’t have any children had more unmet needs in several items of psychological needs, physical and daily living needs and health system and information needs. Patients undergoing chemotherapy had more unmet needs in all domains, especially in psychological needs.

### Factors associated with unmet needs

We compared sociodemographic, medical and psychological factors between patients with and without unmet needs in univariate analyses. The factors that were significantly correlated with the presence of unmet needs were: change in income status after a cancer diagnosis (decreased) (*p* = 0.001); change in work/school life after a cancer diagnosis (somewhat changed) (*p* < 0.001); PS (*p* < 0.001); chemotherapy (yes) (*p* = 0.003); social support (*p* = 0.003).

Using these significant correlated factors in univariate analysis and the associated factors (sex, age, age of first diagnose and marital status) which were reported in previous research, we conducted a multiple regression analysis to identify independent associated factors for unmet needs (Table 3). The results revealed that PS, change in work/school life after a cancer diagnosis and poor social support were associated with total needs. PS was associated with unmet needs of all domains. Poor social support was associated with unmet psychological needs, health system and information needs, and care and support needs. Patients who had experiences change in work/school life after a cancer diagnosis were more likely to have unmet supportive care needs except for sexuality needs.

**Table 3:** Associated factors of unmet needs – multiple regression analysis

	**Total needs**				**Physical & daily living needs**				**Psychological needs**			
	B	β	t	*p*	B	β	t	*p*	B	β	t	*p*
PS (0, 1, 2, 3, 4)	19.401	0.313	4.723	< 0.001	3.499	0.409	6.686	< 0.001	5.514	0.280	4.252	< 0.001
Change in work & school life (Ref: yes)	−14.622	−0.190	−2.889	0.004	−2.916	−0.275	−4.491	< 0.001	−5.873	− 0.240	−3.676	< 0.001
Social support	−0.561	− 0.144	− 2.267	0.024	–	–	–	–	− 0.184	− 0.148	− 2.356	0.019
	**Health system & information needs**				**Care and support needs**				**Sexuality needs**			
	B	β	t	*p*	B	β	t	*p*	B	β	t	*p*
PS (0, 1, 2, 3, 4)	6.279	0.263	3.764	< 0.001	2.804	0.266	3.858	< 0.001	1.406	0.247	3.647	< 0.001
Change in work & school life (Ref: yes)	3.725	0.125	1.771	0.078	−1.667	−0.128	−1.862	0.064	–	–	–	–
Social support	−0.189	− 0.126	−1.888	0.060	− 0.088	− 0.133	−2.018	0.045	–	–	–	–

B: Coefficient, β: Standardized coefficient

A reference is provided for a categorical variable. A low score for a social support scale represents poor social support

The total score of the SCNS-SF34 was significantly associated with both psychological distress (K6 total: *r* = 0.608, *p <* 0.001) and QOL (EQ-5D-5L: *r* = 0.618, *p <* 0.001).

## Discussion

To our knowledge, this study is the first report to assess unmet supportive care needs among Asian young adult cancer patients. Miyashita et al. [22] reported unmet information needs and they were related to QOL in young breast cancer survivors (18–45 years of age at diagnosis and 21–64 years of age at the time of the survey) in Japan. Satisfaction with overall communication with medical professionals was the most unmet information need, which was related to QOL. Those results and our study may lay a foundation for future intervention studies for Japanese young adults with cancer. Our findings are consistent with previously reported findings [23–25]. Sender et al. [23, 24] found that young adult cancer patients most often reported their psychological supportive care needs. Hall et al. [25] showed the top five moderate/high needs reported by young adult cancer survivors. Those items were ‘Fears about the cancer spreading’, ‘Uncertainty about future’, ‘Concerns about the worries of those close to you’, ‘Anxiety’ and ‘Worry that treatment is beyond your control’ in the psychological domain, and ‘Lack of energy’ in the physical and daily living domain. They were also in the top ten unmet needs and seven items of the top ten unmet needs belonged to the psychological domain in our study. Unmet psychological needs of young adult cancer patients are as high as Japanese patients over 40 years of age [26, 27], and should be examined.

Poor social support was associated with higher levels of unmet total supportive care needs and three domains. A cross-sectional study has demonstrated that AYAs with cancer report greater challenges in social functioning compared with the general AYA population [8]. AYAs with cancer frequently report difficulties in maintaining or making new social relationships because of the long-term effects of treatment or feeling anxious concerning fitting into their peer group again [28, 29]. Husson et al. [30] found that AYAs with cancer who had low social functioning reported more physical symptoms and higher levels of distress, and they perceived themselves to receive less social support. We also found that single patients and patients who don’t have any children had more unmet needs in several items of psychological needs, physical and daily living needs, and health system and information needs. Social support and social functioning are key issues in terms of development of supportive care programs and services.

Our finding that patients who had experiences of change in work/school life after a cancer diagnosis was more likely to have unmet supportive care needs is also important and there is no study that reports association between unmet needs and experience of change in work/school life after a cancer diagnosis. Previous studies showed that work-related issues resulted in distress [31], and the ability to return or to maintain occupational and educational pursuits after a cancer diagnosis has been demonstrated to improve the QOL of patients with cancer, reducing social isolation and increasing self-esteem [32, 33]. Work-related issues are one of the major topics in studies on cancer survivors. It is necessary for employers, companies and schools to understand their situations and cooperate. As the Japanese government has been focusing on improving the circumstances of the working population and children [34], it is expected to improve their occupational and educational status. Though higher levels of unmet supportive care needs was associated with higher psychological distress and lower QOL in this study, it might be possible to improve psychological distress and QOL by maintaining occupational and educational status after a cancer diagnosis among young adults with cancer.

Although the number of patients undergoing chemotherapy was small, we examined the relationship between the presence or absence of chemotherapy and unmet supportive care needs as an exploratory study. Their unmet supportive care needs were much higher than those without chemotherapy. This finding suggests that healthcare professionals need to be more careful if patients undergoing chemotherapy have physical, psychological or information problems. The association between other treatments and supportive care needs could not be examined, because there was no data on other treatments. Gupta et al. [35] found that patients who had completed active cancer treatment tended to rate receiving information about diagnosis, cancer and fertility as more important than those who were on active treatment. However, service needs were similar in both groups. Few studies have examined the differences in needs between patients under treatment, those who have completed treatment, and the type of treatment. Further research is needed as supportive care needs could change during the transition process from active treatment to survivorship [16].

This study has some limitations. First, we didn’t have data on participation rate or refusal rate, because a web-based survey company recruited potential participants from a pool of registered people and we got the data from those who responded with interest. However, this study enabled a national survey among young adults with cancer in spite of relatively low prevalence. Second, there is a gender imbalance in that more women than men participated. According to the data of the Cancer Registry of Japan in 2016 and 2017, there are more female than male cancer patients over 20 years old and 80% of patients in the 20–39-year-old group are female [36]. Furthermore, the male-female ratio of registrants aged between 20 and 39 during the study period was approximately 1:2, and more female than male cancer patients were registered in the database (75.6%; 776 female). A gender imbalance in our study might occur due to differences in cancer incidence and gender differences in registrants. Third, 40% of the participants had uterine cancer. According to the data of the Cancer Registry of Japan, the prevalence of uterine cancer in patients aged 20–29 is 9%, and that in patients aged 30–39 is 13% [36]. It is unclear why the study participants had more uterine cancer compared to prevalence. From the distribution of cancer sites in the participants, it is considered that there may be a selection bias. We should be careful about generalizing the results. Fourth, there was no participant aged under 20 years probably because parents’ consent was needed in order to participate this survey if they were under 20 years old. It is necessary to clarify unmet needs among Japanese adolescents with cancer by other methods. Finally, the cross-sectional design provides no information on causal relationships.

## Conclusions

This survey demonstrated that 76.7% of Japanese young adult patients with cancer reported having at least one unmet supportive care needs. The prevalence was relatively high in the psychological domain and low in the sexual domain. In a multiple regression analysis, perceived poorer PS, experience of change in work/school life after a cancer diagnosis and poor social support were associated with total needs. The findings suggest potential opportunities for intervention in addressing psychological needs rather than physical and information needs. And our study would lay a foundation for future intervention studies to improve their psychological adjustment and QOL.

## Data Availability

The datasets used and analyzed during the current study are available from the corresponding author on reasonable reason.

## Abbreviations

AYAs: Adolescence and young adults
PS: Performance status
QOL: Quality of life
